# Reviews of Dental Literature

**Published:** 1901-09

**Authors:** 


### Reviews of Dental Literature.

The Prevention of Nausea and Vomiting during Anæsthesia.—In the *New York Medical Journal* of December 15, 1900, Hirschman reminds us that one of the most annoying things in connection with the administration of chloroform and ether as general anæsthetics is the almost invariable nausea and vomiting produced by their inhalation. The patient, if a person of extremely nervous temperament, is usually nauseated when the administration of the anæsthetic is stated; or, if he is a person of strong and robust build, during the stage of excitement. He may escape, however, until the operation is well under way, when, if allowed to come out of the anæsthetic for any reason whatever, he invariably begins to gag and usually to vomit. In quite a number of cases there is no trouble until the patient is returned to his bed, and allowed to recover from the influence of the anæsthetic, when repeated attempts to vomit sometimes undo the good work of the surgeon or gynæcologist.

The new drug, chloretone, is a very valuable hypnotic, antiseptic, and analgetic. A one per cent. aqueous solution is said to be equal to a two per cent. solution of cocaine hydrochloride for local anæsthesia. Chloretone has the advantage over cocaine of having antiseptic properties, and it has no bad systemic effects. As an hypnotic, it seems to act upon the central nervous system, and in doses of eight to twenty grains (a drachm if necessary) it has a most excellent effect; it is not a depressant to the circulatory and respiratory systems. Dr. Hirschman mentions these uses of the drug incidentally merely as a matter of general knowledge, born of actual experience while he was connected with the Harper Hospital and since. The subject of this paper, however, demands the discussion of the use of chloretone in connection with the prevention of the nausea and vomiting occurring during anæsthesia.

The proposition which presented itself to the writer was this: If vomiting at the beginning of and during the anæsthesia is reflex, and if the vomiting following is due partially to reflex action and largely to the swallowing of the supersecretion of mucus resulting from the action of the anæsthetic on the mucous membranes, why should not a drug which is not a depressant, which is both a local anæsthetic, with pronounced effect on mucous membranes, and a sedative to the central nervous system, be used before anæsthesia to prevent the coincident gastric disturbances?

In accordance with this line of thought, while he was a member of the resident staff of the Harper Hospital, Hirschman began giving chloretone to patients before anæsthetizing them. It was givin in doses of ten grains to women and boys under sixteen years of age, and of fifteen grains to men, half an hour before the anæsthetic was to be started. If the patient does not object to it, he prefers giving it dry on the tongue, and following the administration with an ounce or two of warm water. It may also be given in capsules; but the three-grain sugar-coated tablets now on the market are not so desirable in this connection, because of the number of tablets necessary to make the required dose, and because the sugar coating and compression delay absorption somewhat. When the time for administering the anæsthetic comes around, the patient who has had chloretone is calm; nervousness is quieted, and he is in an ideal condition. If a female, she takes the anæsthetic well, takes little of it, and goes under without a murmur, and one almost forgets that there ever was a stage of excitement.

It takes from seven to twelve minutes to completely anæsthetize with chloroform, and up to fifteen and eighteen with ether. Patients who have had chloretone require less of the anæsthetic, and are not so apt to come out of the anæsthesia suddenly during the operation, if the administration of the anæsthetic is lessened temporarily.

Hirschman personally has observed sixty cases in a space covering a little over three weeks. Half of the patients received chloretone, and the other half, for the purpose of comparing results, did not. The operations were of the same class, the operators were the same, and he personally gave the anæsthetic in nearly all cases. The amount of anæsthetic in the cases of patients receiving the chloretone was from one-third to one-half less than in those not receiving it, and in none of the thirty receiving the drug was stimulation required during the anæsthesia. Out of the thirty receiving chloretone, none was nauseated while being anæsthetized, and three, or ten per cent., were nauseated, and only one of these vomited more than twice on coming out from under the influence of the anæsthetic. Of the thirty less fortunate patients who did not receive chloretone, twenty-four, or eighty per cent., were nauseated and vomited, and nineteen of them were still unable to retain liquid food on the following day; in some of these the nausea and vomiting persisted at intervals for three or four days. The benefits of chloretone in this connection are self-evident. The difference of seventy per cent. in its favor is the most powerful argument for it.

These thirty cases, taken in direct succession, were mostly gynæcological; on account of their sex these individuals were naturally more prone to reflex nervous disturbances. It was in the hope of overcoming this reflex irritability that he sought for a remedy, or, to speak more accurately, a prophylactic. In the cases under observation, chloroform was the anæsthetic usually chosen and always preferred; ether was given only when chloroform, for some reason or other, was contraindicated, or the operation was unduly prolonged.—*Therapeutic Gazette.*

